# Endogenous GDNF Is Unable to Halt Dopaminergic Injury Triggered by Microglial Activation

**DOI:** 10.3390/cells13010074

**Published:** 2023-12-29

**Authors:** Julieta Mendes-Oliveira, Filipa L. Campos, Susana A. Ferreira, Diogo Tomé, Carla P. Fonseca, Graça Baltazar

**Affiliations:** 1 CICS-UBI—Health Sciences Research Centre, University of Beira Interior, 6201-506 Covilhã, Portugal; 2 Faculty of Health Sciences, University of Beira Interior, 6201-506 Covilhã, Portugal

**Keywords:** astrocytes, conditioned media, crosstalk, dopaminergic neurons, GDNF, microglia, neuroinflammation, neuroprotection, Parkinson’s disease, soluble factors

## Abstract

Overactivation of microglial cells seems to play a crucial role in the degeneration of dopaminergic neurons occurring in Parkinson’s disease. We have previously demonstrated that glial cell line-derived neurotrophic factor (GDNF) present in astrocytes secretome modulates microglial responses induced by an inflammatory insult. Therefore, astrocyte-derived soluble factors may include relevant molecular players of therapeutic interest in the control of excessive neuroinflammatory responses. However, in vivo, the control of neuroinflammation is more complex as it depends on the interaction between different types of cells other than microglia and astrocytes. Whether neurons may interfere in the astrocyte-microglia crosstalk, affecting the control of microglial reactivity exerted by astrocytes, is unclear. Therefore, the present work aimed to disclose if the control of microglial responses mediated by astrocyte-derived factors, including GDNF, could be affected by the crosstalk with neurons, impacting GDNF’s ability to protect dopaminergic neurons exposed to a pro-inflammatory environment. Also, we aimed to disclose if the protection of dopaminergic neurons by GDNF involves the modulation of microglial cells. Our results show that the neuroprotective effect of GDNF was mediated, at least in part, by a direct action on microglial cells through the GDNF family receptor α-1. However, this protective effect seems to be impaired by other mediators released in response to the neuron-astrocyte crosstalk since neuron-astrocyte secretome, in contrast to astrocytes secretome, was unable to protect dopaminergic neurons from the injury triggered by lipopolysaccharide-activated microglia. Supplementation with exogenous GDNF was needed to afford protection of dopaminergic neurons exposed to the inflammatory environment. In conclusion, our results revealed that dopaminergic protective effects promoted by GDNF involve the control of microglial reactivity. However, endogenous GDNF is insufficient to confer dopaminergic neuron protection against an inflammatory insult. This reinforces the importance of further developing new therapeutic strategies aiming at providing GDNF or enhancing its expression in the brain regions affected by Parkinson’s disease.

## 1. Introduction

Glial cell line-derived neurotrophic factor (GDNF) was originally purified in 1993 from a rat glial cell line as a potent neurotrophic factor for dopaminergic (DA) neurons [1]. Since then, in vitro and in vivo studies recognized the potential of GDNF as a protective and restorative factor for DA neurons and, consequently, as a promising therapeutic agent in Parkinson’s disease (PD) [2,3,4,5,6,7].

PD is associated with gradual and selective degeneration of the nigrostriatal DA neurons, and although the cause of the progressive DA cell loss remains unknown, dysregulation in the activation of microglial cells has been implicated in the process [8,9]. Overactivated microglia release large amounts of reactive nitrogen and oxygen species and pro-inflammatory cytokines that, in excess, damage DA neurons and contribute to the progressive DA neuron death that occurs in PD. On the other hand, injured DA neurons release molecules such as α-synuclein, neuromelanin, and matrix metalloproteinase 3, which in turn promote or potentiate microglial activation and, consequently, its neurotoxic effect [2,4,7]. In PD brains, there is an early increase in microglial activation associated with the loss of nigrostriatal DA terminals that remain throughout the DA degenerative process [10,11,12]. Also, augmented pro-inflammatory cytokines were found in the striatum of PD patients [13].

Some studies have suggested that GDNF, in addition to its neuroprotective role, may modulate microglia reactivity. GDNF heterozygous (Gdnf^+/−^) middle-aged mice possess fewer tyrosine hydroxylase (TH) positive neurons in the substantia nigra, and present increased expression of pro-inflammatory enzymes, namely cyclooxygenase 2 [14]. Moreover, young Gdnf^+/−^ mice show a reduction in striatal TH immunoreactivity that parallels the increase of activated microglia in the substantia nigra. Moreover, after exposure to a DA toxin, these heterozygous mice present an exacerbated decrease in striatal TH immunoreactivity and a greater increase in nigral-activated microglia when compared to wild-type mice [15]. Xing et al. [16], using cortex-striatum-midbrain organotypic cultures, showed that exogenous GDNF administration reduces microglial activation and can protect nigral DA neurons from death triggered by the pro-inflammatory agent lipopolysaccharide (LPS). Altogether, these studies raise the hypothesis that the anti-inflammatory action of GDNF may also contribute to its neuroprotective effect in DA neurons. However, a direct relationship between this dual effect of GDNF has not been clearly demonstrated.

It has been published in the literature that not only exogenous GDNF but also endogenous GDNF, released by astrocytes, is able to inhibit the release of reactive oxygen species (ROS) and phagocytosis promoted by Zymosan A in midbrain microglial cells [17]. However, those studies were performed in a simplified in vitro cellular model. In vivo, the control of neuroinflammation involves the crosstalk between different types of cells, namely astrocytes, microglia, and neurons. Therefore, it is pertinent to investigate the anti-inflammatory effects of endogenous GDNF in a more complex cellular model, closest to the in vivo environment, to infer if endogenous GDNF, derived by astrocytes, is able to control microglial reactivity and, more importantly, provide DA protection.

Therefore, with the present work, we aimed to (1) disclose whether endogenous GDNF, derived from astrocytes, is still able to modulate microglial activation and provide DA protection against an inflammatory stimulus in a more complex in vitro model where interactions between neurons, astrocytes and microglia are present; (2) determine whether injured DA neurons may interfere with the ability of endogenous GDNF to control microglial reactivity; (3) clarify whether the modulation of microglial activation by GDNF contributes to its DA neuroprotective effect.

## 2. Materials and Methods

### 2.1. Cell Cultures and Treatments

All procedures were approved by the Animal-Welfare body and followed the requirements of the European Convention for the Protection of Vertebrate Animals Used for Experimental and Other Scientific Purposes (Directive 2010/63/EU). The rat colony was raised from Wistar Han IGS rats purchased from Charles River (RRID: RGD_2308816). The animals were bred in the animal house facilities, with free access to water and pellet food, under standard humidity and temperature conditions, at a 12 h light-dark cycle. Females (220–260 g) were housed in groups of four in individually ventilated cages.

#### 2.1.1. Ventral Mesencephalic Neuronal and Neuron-Astrocyte Mixed Cultures

Neuronal and neuron-astrocyte mixed cultures from the ventral mesencephalon were prepared as previously described by Bessa et al. [18] with some alterations. Briefly, pregnant female Wistar rats were anesthetized with ketamine (87.5 mg/kg, Sigma-Aldrich, St. Louis, MO, USA, Cat# K-002) and xylazine (12 mg/kg, Sigma-Aldrich, St. Louis, MO, USA, Cat# X1126). The abdominal cavity was opened, and the embryos with 15–16 days were removed from females through an abdominal incision and transferred to iced phosphate-buffered saline (PBS: 140 mM NaCl, 2.7 mM KCl, 1.5 mM KH_2_PO_4_ and 8.1 mM Na_2_HPO_4_, pH 7.2). The region corresponding to the ventral mesencephalon was dissected, stripped of the meninges, and mechanically dissociated in PBS. The resulting cell suspension was centrifuged at 405× *g* for three minutes and resuspended in Neurobasal Medium (21103-049, Gibco, Paisley, Scotland) supplemented with 2% B27 (17504-044, Gibco, Paisley, Scotland), 0.5 mM L-glutamine (G3126, Sigma-Aldrich, St. Louis, MO, USA), 25 µM L-glutamic acid (G8415, Sigma-Aldrich, St. Louis, MO, USA) and 120 µg/mL gentamicin solution (G1272, Sigma-Aldrich, St. Louis, MO, USA). To obtain a mixed culture of neurons and astrocytes, 10% fetal bovine serum (S0615, Biochrom, Berlin, Germany) inactivated by heat (56 °C for thirty minutes) was added to the culture medium. The culture medium of neuronal cultures was not supplemented with fetal bovine serum. The cells were plated at a density of 2.11 × 10^5^ cells/cm^2^ in poly-D-lysine (P1024, Sigma-Aldrich)-coated plates and maintained at 37 °C in a 5% CO_2_ and 95% air atmosphere.

Immunocytochemistry analysis of neuronal cultures showed that 98.1% of the total number of cells in culture were positive for Microtubule-Associated Protein 2 (MAP2, neuron marker), and 0.6% were positive for Glial Fibrillary Acidic Protein (GFAP, astrocyte marker) (Figure S1). A similar analysis of the neuron-astrocyte mixed cultures showed that 92.0% of the total number of cells in the culture were positive for MAP2, while 5.9% were positive for GFAP (Figure S2).

#### 2.1.2. Ventral Midbrain Glial Cell Cultures

The ventral midbrain of Wistar rat pups (postnatal days 2–5) was dissected, stripped of the meninges, and transferred to iced PBS. After mechanical dissociation in M10C-G medium (Minimum Essential Medium Eagle (MEM, M0268, Sigma-Aldrich, St. Louis, MO, USA) containing 2.2 g/L NaHCO_3_ (S/4240/60, Fisher Scientific, Loughborough, UK), pH 7.3, and supplemented with 10% fetal bovine serum (S0615, Biochrom, Berlin, Germany), 12 U/mL penicillin plus 12 µg/mL streptomycin (A 2213, Biochrom, Berlin, Germany), 3.4 g/L D-glucose anhydrous (G/0450/60, Fisher Scientific, Loughborough, UK) and 5 mg/L insulin from bovine pancreas (I5500, Sigma-Aldrich, St. Louis, MO, USA)), the resulting cell suspension was filtered through a 70 µm mesh and centrifuged at 405× *g* for three minutes. The cells were then resuspended in M10C-G and plated at a density of 9.25 × 10^4^ cells/cm^2^ in poly-D-lysine (P1024, Sigma-Aldrich, St. Louis, MO, USA)-coated plates. After approximately two hours and a half, the cells were washed with cold MEM to remove neurons, and glial cells were cultured in M10C-G medium at 37 °C in an atmosphere of 5% CO2 and 95% air until confluency was achieved.

Immunocytochemistry analysis of these cultures, when the astrocyte-conditioned media was prepared (as described in Section 2.1.6), showed that less than 1% of the cells in the culture were positive for Iba-1 (microglia marker) [17].

#### 2.1.3. Ventral Midbrain Microglia Cultures

When glial cell cultures were confluent, astrocytes were removed by mild trypsinization (0.125 g/L trypsin from porcine pancreas (T7409, Sigma-Aldrich, St. Louis, MO, USA) and 0.05 g/L EDTA (131669, Panreac, Darmstadt, Germany) in MEM). The adherent microglial cells were maintained in culture, in M10C-G medium, for a further five to seven days until use. Immunocytochemistry analysis showed that about 97.9% of the cells in the culture were positive for ionized calcium-binding adaptor molecule 1 (Iba-1) (Figure S3).

#### 2.1.4. Neurons, Astrocytes, and Microglia Co-Cultures

The co-culture system consisted of two different cell cultures maintained in contact through the culture medium. For this, the mesencephalic cultures of neurons and astrocytes were plated in coverslips containing small paraffin spheres, while ventral midbrain microglia cultures were cultured adherent to wells. When both cultures were ready to use, coverslips with neurons and astrocytes, or with neurons only, were transferred to the wells containing the microglial cells. The paraffin spheres ensured the physical separation between the two cell cultures.

#### 2.1.5. Secretome from Neuron-Astrocyte Mixed Cultures

After three days in culture, the medium of neuron-astrocyte mixed cultures was changed to non-essential amino acid medium, consisting of MEM (M0268, Sigma-Aldrich, St. Louis, MO, USA) with 2.2 g/L NaHCO_3_ (S/4240/60, Fisher Scientific, Loughborough, UK), pH 7.3, and supplemented with 1 g/L D-glucose anhydrous (G/0450/60, Fisher Scientific, Loughborough, UK), 0.292 g/L L-glutamine (G3126, Sigma-Aldrich, St. Louis, MO, USA), 0.11 g/L pyruvic acid (P2256, Sigma-Aldrich, St. Louis, MO, USA), 1% MEM non-essential amino acid solution (M7145, Sigma-Aldrich, St. Louis, MO, USA) and 10% heat-inactivated fetal bovine serum (S0615, Biochrom, Berlin, Germany). The choice of this medium was based on its compatibility with neurons, astrocytes, and microglial cells. Twelve hours later the cells were exposed to 1-methyl-4-phenylpyridinium neurotoxin (MPP^+^, 10 µM; D048, Sigma-Aldrich, St. Louis, MO, USA). After thirty-six hours, the conditioned media (CM) were collected from both control neuron-astrocyte cultures (NA-CM) and neuron-astrocyte cultures exposed to MPP^+^ (NA(MPP^+^)-CM) and stored at −80 °C until use.

#### 2.1.6. Secretome from Astrocyte Cultures

Five days after the glial cell culture, the medium was changed to a non-essential amino acid medium. Thirty-six hours later, the media conditioned by astrocytes (A-CM) was collected and stored at −80 °C until use.

#### 2.1.7. Treatments of Ventral Midbrain Microglial Cells

Five to seven days after trypsinization, the different CM (NA-CM, NA(MPP^+^)-CM and A-CM) or GDNF (100 pg/mL, sc-4865, Santa Cruz Biotechnology, Dallas, TX, USA) were added to the microglia cultures. Twenty-four hours later, the cells were exposed to LPS (0.1 or 2 µg/mL, L4391, Sigma-Aldrich, St. Louis, MO, USA) for a further twenty-four hours. The supernatants were then collected for nitric oxide (NO) measurement, and the cells were used to evaluate phagocytic activity.

#### 2.1.8. Treatments of Neurons and Microglia Co-Cultures

Five days after the preparation of mesencephalic neurons and five to seven days after obtaining the microglia by trypsinization, the two cultures were placed in contact as previously described. Approximately seven hours before being placed in contact, the medium of the two cultures was changed to a non-essential amino acid medium. Two hours after the two cultures were placed in contact, the different CM or GDNF (100 pg/mL, sc-4865, Santa Cruz Biotechnology) were added to the co-culture. Twenty-four hours later, the cells were exposed to LPS (10 µg/mL, L4391, Sigma-Aldrich, St. Louis, MO, USA) for a further twenty-four hours. The coverslips with the neurons were removed, fixed with 4% paraformaldehyde, and then processed for TH immunocytochemistry.

#### 2.1.9. Treatments of Neurons and GFRα-1 Silenced Microglia Co-Cultures

Three days before placing neurons and microglia in contact, at the end of the day, the medium of ventral midbrain microglia cultures was replaced by M10C-G without fetal bovine serum or antibiotic. The next morning, the GDNF family receptor α-1 (GFRα-1) was knocked down using a solution containing 72 nM GFRα-1 siRNA (sc-270400, Santa Cruz Biotechnology, Dallas, TX, USA) and 1.9 µL siRNA transfection reagent (sc-29528, Santa Cruz Biotechnology, Dallas, TX, USA) in 300 µL siRNA Transfection Medium (sc-36868, Santa Cruz Biotechnology, Dallas, TX, USA). Six to seven hours later, 300 µL M10C-G containing fetal bovine serum and antibiotic were added to the culture. Approximately twenty-four hours later, the medium of the two cultures was changed to a non-essential amino acid medium. The neurons at five days in vitro were then put in contact through the medium with GFRα-1 siRNA-transfected microglia obtained by trypsinization five to seven days before. Two hours later, GDNF (100 pg/mL, sc-4865, Santa Cruz Biotechnology, Dallas, TX, USA) was added to the co-culture, and twenty-four hours later, the culture was exposed to LPS (10 µg/mL, L4391, Sigma-Aldrich, St. Louis, MO, USA). After twenty-four hours, the coverslip with the neurons was removed from the co-culture, fixed with 4% paraformaldehyde, and processed for TH immunocytochemistry.

#### 2.1.10. Treatments of Neurons, Astrocytes, and Microglia Co-Cultures

Four to five days after preparing the neuron-astrocyte culture and five to seven days after obtaining the microglia by trypsinization, the two cultures were placed in contact through the medium and incubated with 5-fluorodeoxyuridine (FDU: uridine 68 μM and 5-Fluoro-5′-deoxyuridine 27 μM). The medium of the two cultures was changed to a non-essential amino acid medium at least seven hours before. Two hours after the two cultures were placed in contact, GDNF (100 pg/mL, sc-4865, Santa Cruz Biotechnology, Dallas, TX, USA) was added to the co-culture, and twenty-four hours later, the cells were exposed to LPS (50 µg/mL, L4391, Sigma-Aldrich, St. Louis, MO, USA). Twenty-four hours after adding LPS, the neuron-astrocyte culture was fixed with 4% paraformaldehyde and processed for TH immunocytochemistry. Microglial reactivity was monitored by measuring NO levels in the experimental medium.

### 2.2. Phagocytosis Assay

At the end of the incubation period with LPS, microglia cultures were exposed to 0.01% fluorescent microspheres (F13080, Invitrogen, Waltham, MA, USA) in M10C-G for fifteen minutes at 37 °C. After two washes with culture medium, the cells were fixed with 4% paraformaldehyde for twenty minutes and then incubated with 2 µM Hoechst 33342 (H1399, Invitrogen, Waltham, MA, USA) diluted in PBS containing 0.1% Tween-20 (PBS-T) for an additional period of ten minutes. The phagocytic cells were quantified in three independent cell cultures. In each cell culture, three to six coverslips per experimental condition were prepared. In each coverslip, twenty different fields were analyzed. The number of phagocytic cells was normalized to the total number of cells, determined by the quantification of the nuclei stained with Hoechst 33342. The images were acquired with a 63× magnification in an epifluorescence microscope (AxioImager.A1, Zeiss, Jena, Germany).

### 2.3. Determination of NO Release

NO in cell culture supernatants was determined by measuring the total amount of nitrite using the Griess method, as previously described [19]. Nitrite levels were quantified in at least three experiments performed in independent cell cultures. For each experimental condition, two to six replicates were performed.

### 2.4. Measurement of GDNF Levels by ELISA

The levels of GDNF were determined in the different CM using the GDNF Emax^®^ immunoassay system (Promega, Madison, WI, USA), according to the manufacturer’s instructions and as previously described [17]. For each experimental condition, GDNF concentration was measured in two to four replicates from three independent cell cultures.

### 2.5. Immunocytochemistry

Immunocytochemistry was performed as previously described [19]. The cells were incubated overnight at 4 °C with the primary antibodies, selected according to the goal of the experiment: mouse anti-MAP2 antibody (1:500; sc-74421, Santa Cruz Biotechnology, Dallas, TX, USA), rabbit anti-GFAP antibody (1:2000, Z0334, Dako, Santa Clara, CA, USA), mouse anti-GFAP antibody (1:200, MA5-12023, Invitrogen, Waltham, MA, USA), rabbit anti-Iba-1 antibody (1:2000, 019-19741, Wako, Osaka, Japan), and mouse anti-TH antibody (1:1000; 612300, BD Biosciences, San Jose, CA, USA). After primary antibody incubation, the cells were incubated for two hours, at room temperature, with the following secondary antibodies: goat anti-rabbit antibody conjugated to Alexa Fluor 546 (1:1000, A11010, Invitrogen, Waltham, MA, USA), goat anti-rabbit antibody conjugated to Alexa Fluor 488 (1:1000, A11008, Invitrogen, Waltham, MA, USA), and goat anti-mouse antibody conjugated to Alexa Fluor 488 (1:1000, A11001, Invitrogen, Waltham, MA, USA). The cell’s nuclei were stained by incubation for ten minutes with 2 μM Hoechst 33342 (H3569, Invitrogen, Waltham, MA, USA). MAP2-, GFAP-, Iba-1-, and TH-positive cells were quantified in at least three independent cell cultures. For each independent experiment, two to six coverslips per experimental condition were analyzed. The number of GFAP, MAP2, Iba-1, or TH positive cells present in twenty microscope fields were quantified in each coverslip and normalized to the total number of cells obtained by quantification of the nuclei stained with Hoechst 33342. The images were acquired on an epifluorescence microscope AxioObserver Z1 with an AxioCamMR3 camera and EC Plan-Neofluar 40×/1.3 Oil DIC M27 lens. Acquisition and processing of images were made with the AxioVision software (Carl Zeiss MicroImaging GmbH, Munich, Germany, version 4.8.2.0).

### 2.6. Analysis of GFRα-1 Knockdown by Real-Time PCR

The efficacy of GFRα-1 knockdown in microglial cells was analyzed by quantifying GFRα-1 mRNA expression by real-time PCR. Total RNA was extracted from microglial cells exposed to the GFRα-1 siRNA (sc-270400, Santa Cruz Biotechnology, Dallas, TX, USA) or to a control siRNA (sc-37007, Santa Cruz Biotechnology, Dallas, TX, USA) or only to transfection reagent (sc-29528, Santa Cruz Biotechnology, Dallas, TX, USA). RNA concentration and purity were determined by measuring the absorbance at 260 nm and 280 nm (NanoPhotometer, Implen, Munich, Germany). RNA integrity was evaluated by 1% agarose gel electrophoresis. RNA (1 µg) in a 17 µL solution containing 50 ng random primers (MB12901, Nzytech, Lisboa, Portugal) and 0.59 mM deoxynucleotide triphosphates (R0141, R0151, R0161 and R0171, Thermo Fisher Scientific, Vilnius, Lithuania) was denatured at 65 °C for five minutes. For the reverse transcription, this solution was mixed with 3 µL of NZY M-MuLV Reverse Transcriptase (200 U, diluted in the enzyme buffer, MB08301, Nzytech, Lisboa, Portugal). After fifty minutes at 37 °C, reverse transcription was stopped by enzyme inactivation at 70 °C for fifteen minutes. Then, real-time PCR was developed using the iCycler IQTM Real-Time PCR Detection System (Bio-Rad, Hercules, CA, USA). The reactions were performed in a total volume of 20 µL containing 2 µL cDNA, 0.3 µM of each primer, and 10 µL of Luminaris HiGreen Fluorescein qPCR Master Mix (K0983, Thermo Fisher Scientific, Vilnius, Lithuania). An initial denaturation at 95 °C for three minutes was followed by 40 cycles at 95 °C for ten seconds, 56 °C for forty-five seconds, and 72 °C for ten seconds. The Melting Curve was obtained by increasing the temperature from 55 °C to 95 °C with increments of 0.5 °C. GFRα-1 mRNA levels were evaluated using the forward primer: 5′_ACTCCTGCAAGACCAATTACA_3′ and the reverse primer: 5′_CAGTTGCTGACAGACCTTGA_3′ and were normalized to those of cyclophilin A. To assess the cyclophilin A mRNA levels, the following primers were used: 5′_CAAGACTGAGTGGCTGGATGG_3′ (forward) and 5′_GCCCGCAAGTCAAAGAAATTAGAG_3′ (reverse). The results were obtained from four to six independent cell culture experiments and are expressed as 2^−ΔΔCT^ relative to the transfection reagent condition.

### 2.7. Statistical Analysis

Statistical analysis was performed with the GraphPad Prism 8.0.1 using the unpaired *t*-test or the one-way analysis of variance (ANOVA) followed by Dunnett’s Multiple Comparison Test or Bonferroni’s Multiple Comparison Test, as described in the figure legends.

## 3. Results

### 3.1. Neuron-Astrocyte Secretome Modulates Microglial Reactivity but Is Unable to Protect DA Neurons from Injury Triggered by Microglial Activation

It is known that overactivation of microglia may lead to DA neuron injury [20]. Therefore, therapeutic strategies able to modulate microglial reactivity may be therapeutically relevant to protect challenged DA neurons and prevent disease progression. It is known that astrocytes secretome completely inhibits the increase of ROS and phagocytosis induced by an inflammatory stimulus in ventral midbrain microglia [17]. However, in vivo, other cell types are present, namely neurons, that may affect astrocyte’s ability to modulate microglial reactivity. Therefore, the physiological relevance of the anti-inflammatory action of astrocytes was investigated in a more complex cellular system, closer to the in vivo situation, where intercellular communication was enabled between neurons, astrocytes, and microglia. Also, the impact of DA neuron injury on the modulatory action of astrocytes on microglial reactivity was evaluated.

**Figure 1:**
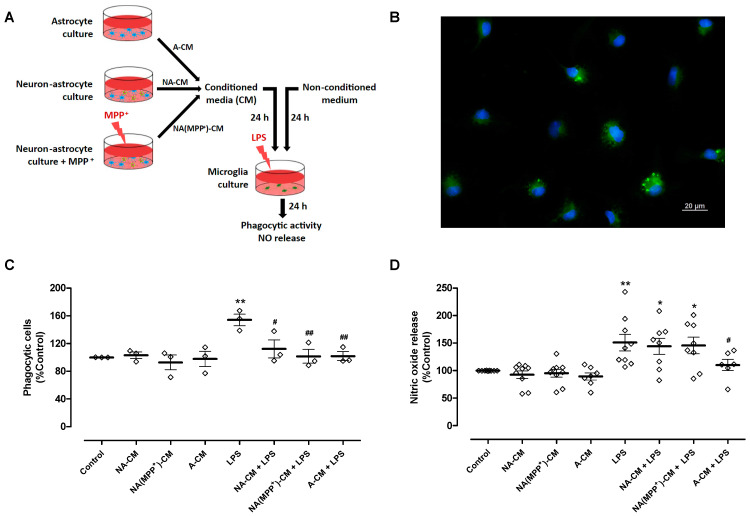
Effect of astrocyte and neuron-astrocyte secretomes on LPS-induced phagocytic activity and NO release in microglial cells. (**A**) Schematic representation of conditioned media collection and treatments of microglia cultures. Ventral midbrain microglia were incubated with a non-conditioned medium (control) and media conditioned by astrocytes (A-CM), neuron-astrocytes (NA-CM), and MPP^+^-exposed neuron-astrocytes (NA(MPP^+^)-CM) cultures, for twenty-four hours, and later exposed to LPS (0.1 µg/mL) for additional twenty-four hours. (**B**) Representative image of microglial cells incubated with green fluorescent microspheres (63× magnification). Cell nuclei were stained with Hoechst 33342 (blue). (**C**) Quantification of microglial cells that engulfed the microspheres (phagocytic cells). (**D**) Measurement of NO released by microglial cells. Each bar represents the mean ± SEM of three (**C**) or six to nine (**D**) independent cell cultures. * *p* < 0.05 and ** *p* < 0.01 compared to control (one-way ANOVA followed by Dunnett’s Multiple Comparison Test); ^#^ *p* < 0.05 and ^##^ *p* < 0.01 compared to LPS (one-way ANOVA followed by Bonferroni’s Multiple Comparison Test).

To achieve these goals, conditioned media from control neuron-astrocyte cultures (NA-CM) and from neuron-astrocyte cultures exposed to the DA toxin MPP^+^ (NA(MPP^+^)-CM) were collected and transferred to ventral midbrain microglia cultures (Figure 1A). MPP^+^, at a concentration of 10 µM, significantly decreased DA neuron viability by 39% after 24 h incubation (61.1 ± 2.4% TH-positive cells (*n* = 12), relative to control (100.3 ± 0.3%, *n* = 12, without MPP^+^)—Figure S4). Media conditioned by astrocytes (A-CM), as well as media not subjected to conditioning, were used as controls. After exposure of microglia to LPS (0.1 µg/mL) for twenty-four hours, in the presence of the different CM, two parameters of microglial activation, phagocytic activity (Figure 1B,C) and NO release (Figure 1D) were analyzed. LPS increased the number of phagocytic cells by 53.9 ± 8.3% when compared to the control, and the A-CM totally inhibited LPS-induced phagocytosis. A similar inhibitory effect was observed both for NA-CM and NA(MPP^+^)-CM (Figure 1C). Concerning NO, as expected, LPS increased the release of NO by 50.9 ± 15.1%, and pre-incubation with A-CM reduced this effect by 40.7 ± 18.2%. However, NA-CM and NA(MPP^+^)-CM were not able to prevent LPS-induced NO release by microglia (Figure 1D). These results show that the crosstalk with neurons interfered, at least in part, with the ability of astrocytes to modulate microglial reactivity since both neuron-astrocyte secretomes (NA-CM and NA(MPP^+^)-CM) were able to prevent the increase in phagocytic activity but not the release of NO by LPS-exposed microglia.

**Figure 2:**
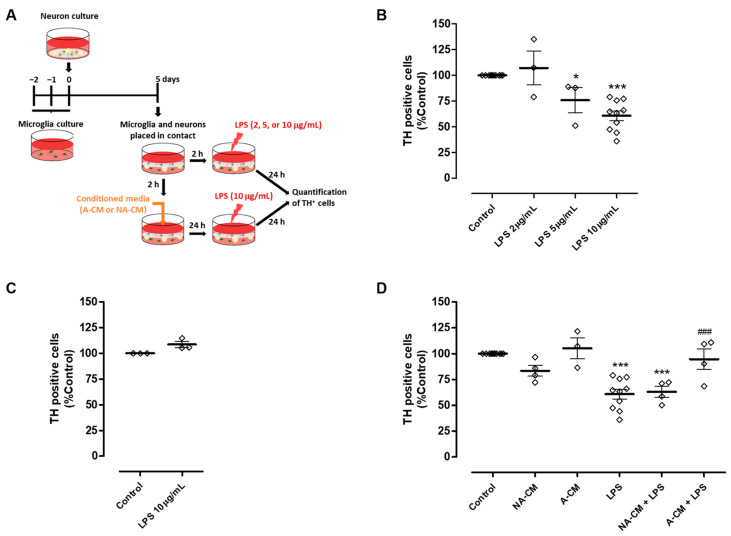
Neuron-astrocyte secretome was unable to protect DA cells against the injury triggered by LPS-induced microglial activation. (**A**) Schematic representation of the preparation of neuron-microglia co-cultures and treatments. Microglia and neuron cultures were placed in contact through the culture medium and then incubated with a non-conditioned medium (control) or media conditioned by astrocytes (A-CM) and neuron-astrocyte (NA-CM) cultures for twenty-four hours and later exposed to LPS (10 µg/mL) for additional twenty-four hours. (**B**) Quantification of TH immunopositive cells in neuron-microglia co-cultures after treatment with increasing concentrations of LPS (2, 5, and 10 µg/mL) for twenty-four hours. (**C**) Quantification of TH immunopositive cells in neuron cultures (in the absence of microglia) upon exposure to 10 µg/mL of LPS for twenty-four hours. (**D**) Quantification of TH immunopositive cells in neuron-microglia co-cultures pre-treated with NA-CM, A-CM, or non-conditioned culture medium (control) and then exposed to 10 µg/mL of LPS for twenty-four hours. Each bar represents the mean ± SEM of three to ten (**B**,**D**) or three (**C**) independent experiments. * *p* <0.05 and *** *p* < 0.001 compared to control (one-way ANOVA followed by Dunnett’s Multiple Comparison Test) and ^###^ *p* < 0.001 compared to LPS (one-way ANOVA followed by Bonferroni’s Multiple Comparison Test).

In the context of Parkinson’s disease, it is important to investigate if this impairment in the modulation of microglial reactivity may have an impact on DA neuron viability when these cells are exposed to an inflammatory environment. Therefore, we explored the ability of neuron-astrocyte secretome to protect DA neurons from injury triggered by microglial activation, using the astrocyte secretome for comparison purposes. To achieve this goal, we implemented a co-culture in which neurons were maintained in contact with microglia through the culture medium. Initially, this co-culture was exposed to different concentrations of LPS to determine the concentration capable of triggering DA injury through microglial activation (Figure 2A). TH immunocytochemistry showed that 10 µg/mL LPS induced a significant reduction of 39.2 ± 4.7% in the number of TH-positive cells (Figure 2B). This reduction was not observed when neurons were directly exposed to 10 µg/mL LPS in the absence of microglia (Figure 2C). The neuron-microglia co-cultures were then incubated with media conditioned by astrocytes, or neurons and astrocytes, for 24 h, followed by exposure to 10 µg/mL LPS for an additional 24 h (Figure 2A). Pre-incubation with astrocyte secretome (A-CM) protected DA neurons from injury induced by LPS-stimulated microglia. On the contrary, pre-incubation with neuron-astrocyte secretome (NA-CM) did not provide protection to DA neurons (Figure 2D).

### 3.2. Supplementation of Neuron-Astrocytes Secretome with Exogenous GDNF Protects DA Neurons from Injury Induced by Microglial Activation

**Figure 3:**
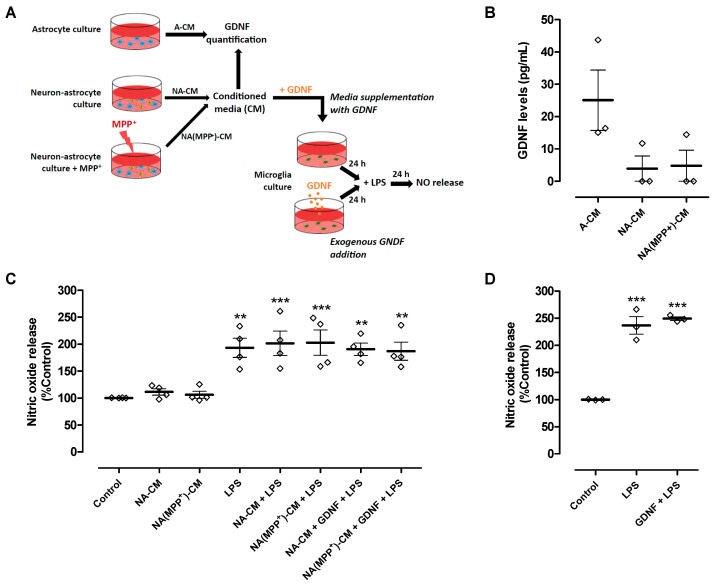
GDNF levels were lower in neuron-astrocyte secretomes compared to astrocyte secretomes, and media supplementation with GDNF or exogenous GDNF addition was unable to reduce LPS-induced NO release by ventral midbrain microglia. (**A**) Schematic representation of conditioned media collection, GDNF quantification in the media, and treatments of microglia cultures with conditioned media supplemented with GDNF or addition of exogenous GDNF directly to the cell cultures. Media conditioned by astrocytes (A-CM), neuron-astrocytes (NA-CM), and MPP^+^-exposed neuron-astrocytes (NA(MPP^+^)-CM) cultures were collected for GDNF quantification. Thereafter, ventral midbrain microglia were incubated with non-conditioned medium (control), NA-CM, and NA(MPP+)-CM supplemented with GDNF (100 pg/mL) for twenty-four hours and later exposed to LPS (0.1 µg/mL) for additional twenty-four hours. In another set of experiments, GDNF (100 pg/mL) was added directly to microglia cultures and incubated for twenty-four hours before the exposure to LPS (0.1 µg/mL) for an additional twenty-four hours. (**B**) Measurement of GDNF levels in media conditioned by astrocytes (A-CM), neuron-astrocytes (NA-CM), and MPP^+^-exposed neuron-astrocytes (NA(MPP^+^)-CM) cultures. (**C**) Measurement of NO released by microglial cells after exposure to LPS (0.1 µg/mL) for twenty-four hours in the presence of NA-CM and NA-(MPP^+^)-CM supplemented or not with GDNF (100 pg/mL) or non-conditioned culture medium (control). (**D**) Measurement of NO released by microglial cells pre-treated with GDNF (100 pg/mL) or non-conditioned culture medium and exposed to LPS (2 µg/mL) for twenty-four hours. Each bar represents the mean ± SEM of three (**B**,**D**) or four (**C**) independent experiments. ** *p* < 0.01 and *** *p* < 0.001 compared to control (one-way ANOVA followed by Dunnett’s Multiple Comparison Test).

Our results showed that the secretome derived from neurons and astrocytes (NA-CM) modulated microglial reactivity induced by LPS but was unable to protect DA neurons from injury, in contrast to astrocyte secretome (A-CM). It is known that astrocyte modulation of microglial reactivity, namely ROS production and phagocytosis, requires the presence of GDNF [17]. Therefore, we hypothesized that when neurons are present, endogenous GDNF may not be sufficient to protect DA neurons, or its action may be counteracted by other soluble factors present in the extracellular medium. Hence, we measured the concentration of GDNF in the neuron-astrocyte and astrocyte CM to infer if their different anti-inflammatory and neuroprotective properties could be due to different levels of GDNF released to the media (Figure 3A). We found that GDNF levels in NA-CM (3.9 ± 3.9 pg/mL) and NA(MPP^+^)-CM (4.8 ± 4.8 pg/mL) were considerably lower than the levels present in the A-CM (25.1 ± 9.3 pg/mL), though statistical significance was not achieved (Figure 3B).

Hence, we determined if supplementing the media with GDNF (100 pg/mL) (Figure 3A) could overcome the inability of neuron-astrocyte secretomes (NA-CM and NA(MPP^+^)-CM) to prevent LPS-induced NO release by microglial cells and, consequently, achieve DA neuronal protection. The results showed that even after adding GDNF at higher levels than those measured in A-CM, the neuron-astrocyte secretomes were incapable of preventing the LPS-induced increase in NO release (Figure 3C). Exogenous GDNF addition, at the same concentration and in the absence of conditioning (Figure 3A), was also unable to decrease LPS-induced NO release by microglia (Figure 3D). These results showed that the inability of neuron-astrocyte secretomes to modulate NO release by LPS-activated microglia was not due to insufficient GDNF levels.

**Figure 4:**
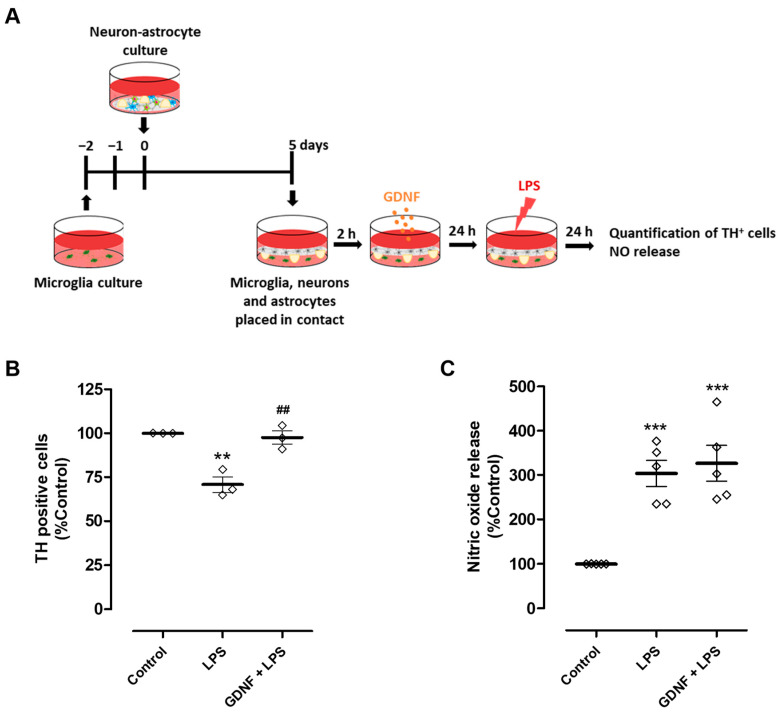
Exogenous GDNF pre-treatment prevents DA neuron loss induced by microglial activation, although it does not prevent NO release by microglia. (**A**) Schematic representation of the preparation of neuron-astrocytes-microglia co-cultures and treatments. Primary neuron-astrocyte culture was placed in contact through the culture medium with microglia and incubated with LPS (50 µg/mL) for twenty-four hours in the presence or in the absence of GDNF (100 pg/mL). (**B**) Quantification of TH immunopositive cells in the neuron-astrocyte culture. (**C**) NO release by microglia, as inferred by the quantification of nitrite levels in cell culture supernatants. Each bar represents the mean ± SEM of three (**B**) or five (**C**) independent experiments. (**B**) ** *p* < 0.01 compared to control and ^##^ *p* < 0.01 compared to LPS (one-way ANOVA followed by Bonferroni’s Multiple Comparison Test); (**C**) *** *p* < 0.001 compared to control (one-way ANOVA followed by Dunnett’s Multiple Comparison Test).

We next investigated the impact of exogenous GDNF supplementation (100 pg/mL) of neuron-astrocyte secretomes on DA neuron viability upon exposure to LPS. To achieve this goal, we used neuron-astrocyte cultures maintained in contact through the medium with microglia cultures (Figure 4A). Due to the presence of astrocytes in these co-cultures and their ability to modulate microglia reactivity [17,21,22,23,24], a higher concentration of LPS was needed to achieve a comparable DA neuron injury extent (50 µg/mL instead of 10 µg/mL used previously in neuron-microglia co-cultures). Exposure to LPS led to a reduction of 29.2 ± 4.4% in the number of TH-positive cells, which was completely inhibited by the pre-treatment with exogenous GDNF (Figure 4B). Therefore, our results show that although exogenous GDNF supplementation of the neuron-astrocyte secretome was unable to prevent LPS-induced NO release by microglial cells (Figure 3C and Figure 4C), it was effective in protecting DA neurons from inflammation-induced injury (Figure 4B).

### 3.3. Microglial GFRα-1 Knockdown Reduces GDNF-Induced DA Protection

**Figure 5:**
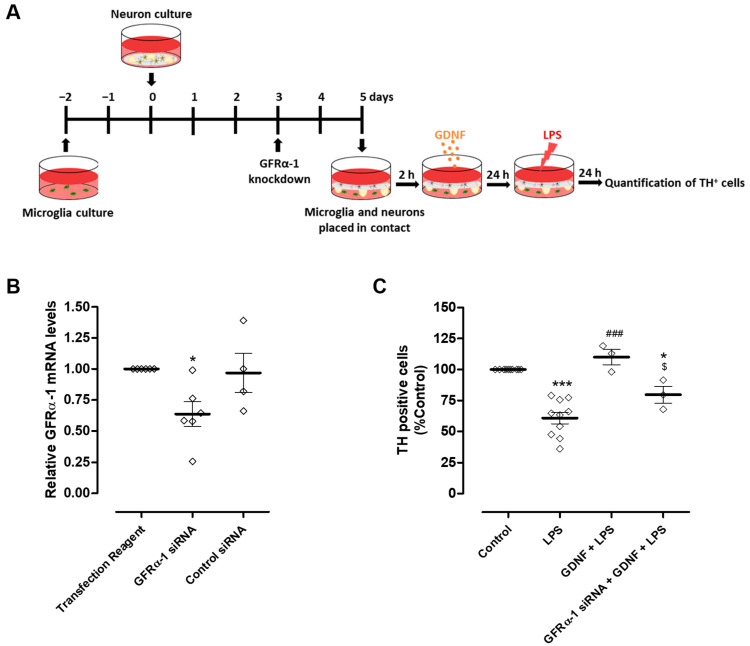
GFRα-1 knockdown in microglial cells decreased the protection of DA neurons promoted by exogenous GDNF upon LPS-induced microglial activation. (**A**) Schematic representation of the preparation of GFRα-1 knockdown microglia (or control microglia)-neurons co-cultures and treatments. GFRα-1 knockdown microglia or control microglia were placed in contact, through the medium, with neurons and incubated with LPS (10 µg/mL) for twenty-four hours in the presence or in the absence of GDNF (100 pg/mL). (**B**) Quantification of microglial GFRα-1 mRNA levels by real-time PCR. (C) Quantification of TH immunopositive cells in neuron culture. Each bar represents the mean ± SEM of four to six (**B**) or three to ten (**C**) different experiments. (**B**) * *p* < 0.05 compared to Transfection Reagent condition (one-way ANOVA followed by Dunnett’s Multiple Comparison), (**C**) * *p* < 0.05 and *** *p* < 0.001 compared to control, ^###^ *p* < 0.001 compared to LPS and ^$^ *p* < 0.05 compared to GDNF + LPS (one-way ANOVA followed by Bonferroni’s Multiple Comparison Test).

Modulation of microglial reactivity by GDNF depends on the activation of GFRα-1 [17]. Therefore, to investigate whether the DA neuron protection exerted by exogenous GDNF supplementation occurs exclusively via a direct effect on these neurons or if it also involves the regulation of microglial activation, we knocked down the GDNF receptor, GFRα-1, specifically in microglial cells (Figure 5A). We achieved a reduction of about 36.4 ± 9.9% in microglial GFRα-1 mRNA levels compared to the transfection reagent condition (Figure 5B). After GFRα-1 knockdown, microglia were placed in contact with neurons through the medium and exposed to LPS (10 µg/mL) in the presence or in the absence of 100 pg/mL GDNF (Figure 5A). Protection of DA neurons exerted by exogenous GDNF was partially reduced by GFRα-1 knockdown in microglial cells from 110 ± 6.3% to 79.6 ± 6.8% TH positive cells (Figure 5C).

## 4. Discussion

Growing evidence suggests that chronic neuroinflammation, mediated by microglial activation, can promote or potentiate DA neurodegeneration in PD [8]. Hence, therapeutic strategies targeting the cellular and/or molecular players in the neuroinflammatory process may be promising to slow down or halt the progression of this disease. Astrocytes regulate the activation of microglial cells, preventing their excessive inflammatory responses and, consequently, their potential neurotoxic effects. In vitro, soluble factors released by astrocytes can reduce the reactivity of microglial cells induced by an inflammatory stimulus, as demonstrated by the reduction in phagocytosis, in the production of inflammatory cytokines, ROS, and NO, and in the expression of inducible nitric oxide synthase (iNOS) [17,21,22,23,24]. Among those soluble factors, astrocyte-derived GDNF plays an important role in the control of microglial responses [17]. The present study confirmed the ability of astrocytes’ secretomes to modulate microglial reactivity as well as to protect DA neurons under pro-inflammatory conditions. However, the control of neuroinflammation in vivo involves intercellular interactions between a variety of cell types, namely microglia, astrocytes, and neurons. We hypothesized that the crosstalk with neurons could influence the anti-inflammatory and neuroprotective actions of astrocytes. Therefore, we implemented a more complex cellular system, in vitro, where communication between these three types of cells was enabled to infer the possible biological relevance of such astrocyte-mediated actions. We also investigated if injured DA neurons could promote or potentiate microglial activation when astrocytes are present since it is known that injured DA neurons release factors such as α-synuclein, neuromelanin, and matrix metalloproteinase 3 that contribute to microglial activation [2,4,7,20].

We found that the crosstalk with either healthy or injured DA neurons did not affect the inhibition of phagocytic activity by astrocytes but interfered with the ability of astrocytes to modulate the release of NO by LPS-stimulated microglia. More importantly, the crosstalk with neurons interfered with the ability of astrocytes to protect DA neurons from injury triggered by an inflammatory insult. Whether insufficient levels of endogenous GDNF in the presence of neurons could be contributing to this impairment was determined. Neither the supplementation of neuron-astrocyte secretome with GDNF (100 pg/mL, a concentration known to inhibit microglial ROS production and phagocytic activity induced by an inflammatory insult [17]) nor exogenous GDNF pre-treatment was able to prevent NO release by reactive microglia upon LPS exposure. In contrast to our results, Rickert et al. [25] demonstrated that pre-treatment of microglia with GDNF could decrease NO release induced by LPS, but the GDNF concentration used was much higher (50,000 pg/mL). This could indicate that the GDNF concentration present in the different neuron-astrocyte secretomes was insufficient to prevent the release of microglial NO triggered by LPS. However, given that the amount of GDNF detected in the three different secretomes was less than 100 pg/mL and that astrocytes’ secretome can still decrease NO levels, it is more likely that other factors released by astrocytes, in addition to GDNF, modulate NO production by microglial cells. For example, transforming growth factor beta (TGF-β) present in the secretome of LPS-stimulated astrocytes can reduce NO release by rat microglia [24]. As this effect was lost in the presence of neurons, we suggest that neurons, or the factors released by them, interfere with the control of microglial NO production/release exerted by astrocytes.

NO is involved in the neurotoxic effect of DA toxins and LPS on DA neurons since inhibition, silencing, or depletion of iNOS promotes DA protection [26,27,28,29,30,31]. Since the secretome collected in the presence of neurons was incapable of decreasing NO release, we assessed its impact on the protection of DA neurons from microglia-induced damage. Exposure of neurons-microglia co-cultures to LPS (10 µg/mL) leads to a significant DA cell loss, an effect due to microglial activation since when LPS alone was added to a neuronal culture in the absence of microglial cells, there was no DA neuron loss, which is in agreement with data from other studies [32,33,34,35,36]. We found that, unlike astrocyte secretome, neuron-astrocyte secretome was not able to protect DA neurons. DA neuroprotection against injury induced by microglial activation was only achieved when exogenous GDNF (100 pg/mL), a well-known neuroprotective agent for DA neurons, was added to the co-culture containing neurons, astrocytes, and microglia. This suggests that the amount of GDNF produced endogenously by astrocytes in the presence of neurons may not be sufficient to protect DA cells from an inflammatory insult and may explain why, under pathological situations, such as in PD, endogenous GDNF seems incapable of protecting DA neurons from progressive degeneration. In agreement with this is the observation that surviving DA neurons in the substantia nigra pars compacta of PD patients contain lower GDNF protein levels than age-matched controls [37]. Also, GDNF signaling seems to be dysregulated in PD [38]. These two impairments, together, significantly decrease the potential of GDNF to exert its anti-inflammatory, neuroprotective, and restorative function in the nigrostriatal DA pathway.

Although we hypothesized that the lower levels of GDNF on NA-CM, compared to A-CM, could be attributed to the presence of neurons in the culture, we cannot exclude that changes in GDNF expression, specifically in astrocytes from different developmental stages, may occur. Particularly, NA-CM was obtained from prenatal cultures, whereas A-CM was prepared from postnatal cultures. However, to the best of our knowledge, there is no evidence in the literature that GDNF expression may vary specifically among astrocytes of different developmental stages.

There are several studies showing that exogenous GDNF mediates protective effects against DA neuronal injury [2,5,6,7,39]. Furthermore, the present results and others [16] show that GDNF can protect DA neurons in an inflammatory environment. To exert its neuroprotective effect, GDNF binds with high affinity to GFRα-1 that, in turn, interacts with the receptor tyrosine kinase (Ret) or, alternatively, with the neural cell adhesion molecule (NCAM) [40]. DA neurons [40] and microglial cells [25,41] express GFRα-1 and Ret, and thus, GDNF can act on both cell types. It has been shown that binding of GDNF to GFRα-1 is required for the modulation of microglial activation by astrocyte-released GDNF [17]. Moreover, a study using GFRα-1 heterozygous (GFRα-1^+/−^) mice found that these animals, besides having less nigral TH positive neurons and lower striatal TH immunoreactivity, presented increased levels of inflammatory markers in the substantia nigra, and that 1-methyl-4-phenyl-1,2,3,6-tetrahydropyridine (MPTP) toxin exacerbated the toxic effects on DA system as compared to wild type mice [42]. To understand whether the regulation of microglial activation could contribute to the protective effect of GDNF on DA neurons, we used a co-culture of neurons and microglia and knocked down GFRα-1 in microglial cells. GFRα-1 knockdown attenuated DA neuron protection promoted by GDNF, showing that the modulation of microglial activation by GDNF significantly contributed to its neuroprotective effect.

## 5. Conclusions

In conclusion, our results show that GDNF exerts its neuroprotective effect on DA neurons through the modulation of microglial activation, in addition to its well-known protective action directly on these neurons. Moreover, they also suggest that endogenous GDNF, when neurons are present, is insufficient to protect DA neurons against an inflammatory insult. Since excessive microglial activation has a key role in the progressive loss of DA neurons in PD and GDNF does not cross the blood–brain barrier, our study reinforces the importance of developing new therapeutic approaches aiming at delivering GDNF more efficiently to the affected brain regions, using brain penetrant GDNF mimetic compounds or boosting the expression of endogenous GDNF in those regions, to protect or restore affected DA neurons in PD.

## Data Availability

The data are contained within the article.

## Supplementary Materials

The following supporting information can be downloaded at https://www.mdpi.com/article/10.3390/cells13010074/s1. Figure S1: Characterization of ventral mesencephalic neuronal cultures. (A) Representative images of immunolabeling for GFAP (red) or MAP2 (green). The nuclei were labeled with Hoechst 33342 (blue). (B) Quantification of GFAP-positive cells (astrocytes) and MAP2-positive cells (neurons) present in the culture, in relation to the total number of cells. The results are expressed as a percentage of neurons and astrocytes normalized in relation to the total number of cells per field and represent the mean ± SEM of three independent cell cultures in which each experimental condition was carried out in duplicate. Statistical analysis was performed using an unpaired *t*-test. **** *p* < 0.0001 compared to the percentage of astrocytes in the culture; Figure S2: Characterization of ventral mesencephalic neuron-astrocyte mixed cultures. (A) Representative images of immunolabeling for GFAP (red) or MAP2 (green). The nuclei were labeled with Hoechst 33342 (blue). (B) Quantification of GFAP-positive cells (astrocytes) and MAP2-positive cells (neurons) present in the culture, in relation to the total number of cells. The results are expressed as a percentage of neurons and astrocytes normalized in relation to the total number of cells per field and represent the mean ± SEM of three independent cell cultures in which each experimental condition was carried out in duplicate. Statistical analysis was performed using an unpaired *t*-test. **** *p* < 0.0001 compared to percentage of astrocytes in the culture; Figure S3: Characterization of ventral midbrain microglia cultures. (A) Representative images of immunolabeling for GFAP (green) or Iba-1 (red). The nuclei were labeled with Hoechst 33342 (blue). (B) Quantification of GFAP-positive cells (astrocytes) and Iba-1-positive cells (microglia) present in the culture, in relation to the total number of cells. The results are expressed as a percentage of microglia and astrocytes normalized in relation to the total number of cells per field and represent the mean ± SEM of three independent cell cultures in which each experimental condition was carried out in quadruplicate. Statistical analysis was performed using an unpaired *t*-test. **** *p* < 0.0001 compared to the percentage of astrocytes in the culture; Figure S4: MPP^+^ (10 µM, 24 h) significantly decreased DA neuron viability in a neuron-astrocyte culture. The cultures were exposed to 10 µM MPP^+^ (or vehicle–control) for 24 h and processed for TH immunoreactivity. The number of TH-positive cells (DA neurons) was quantified and expressed as a percentage of control. The bars represent the mean ± SEM of 12 independent experiments performed in triplicate. Statistical analysis was performed using an unpaired *t*-test. *** *p* < 0.001 compared to the control.
