# Supplementary figures and images for: Endogenous GDNF Is Unable to Halt Dopaminergic Injury Triggered by Microglial Activation
Source: Cells. 2023 Dec 29;13(1):74. doi: 10.3390/cells13010074 (PMC10778367; doi:10.3390/cells13010074)

## Supplementary Figure S1

# A

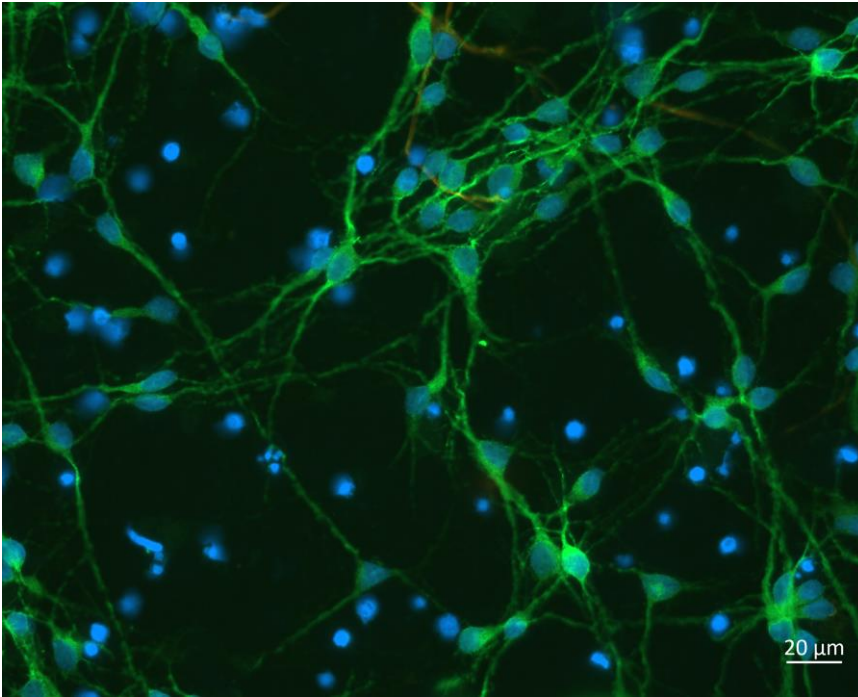

B

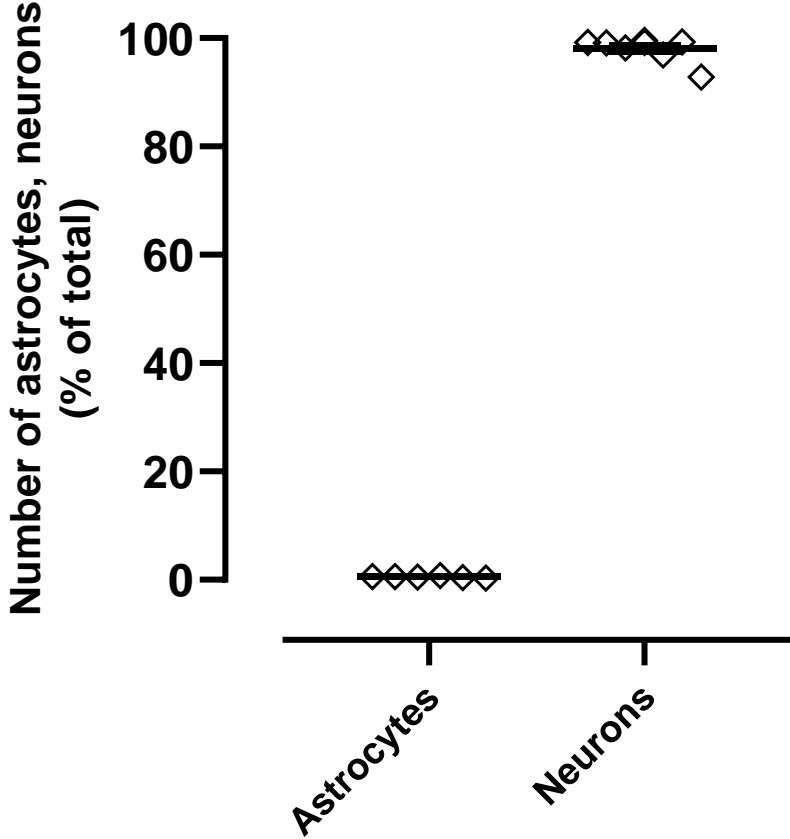

Supplement: Supplementary file 1 [file cells-13-00074-s001.zip › supplementary Figure S1.pdf]

Supplementary Figure S2

A

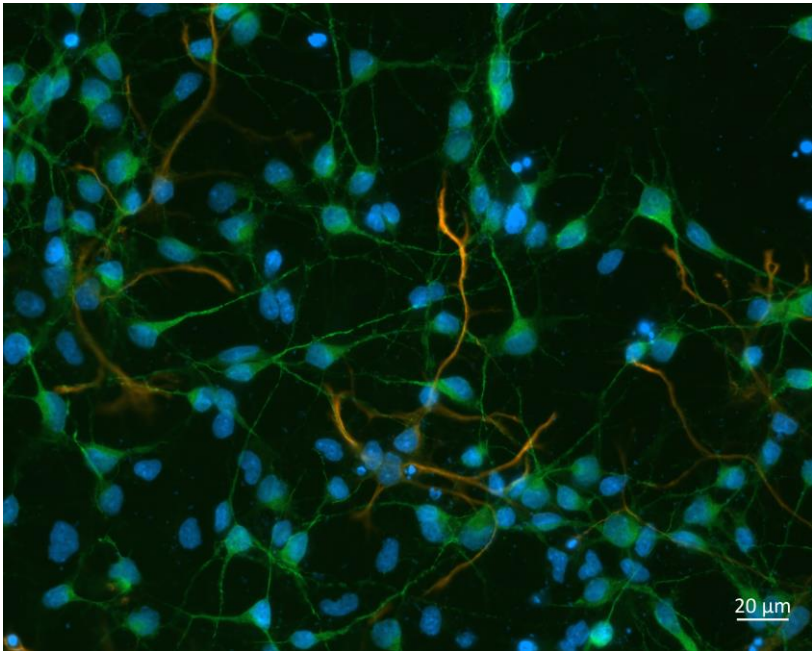

B

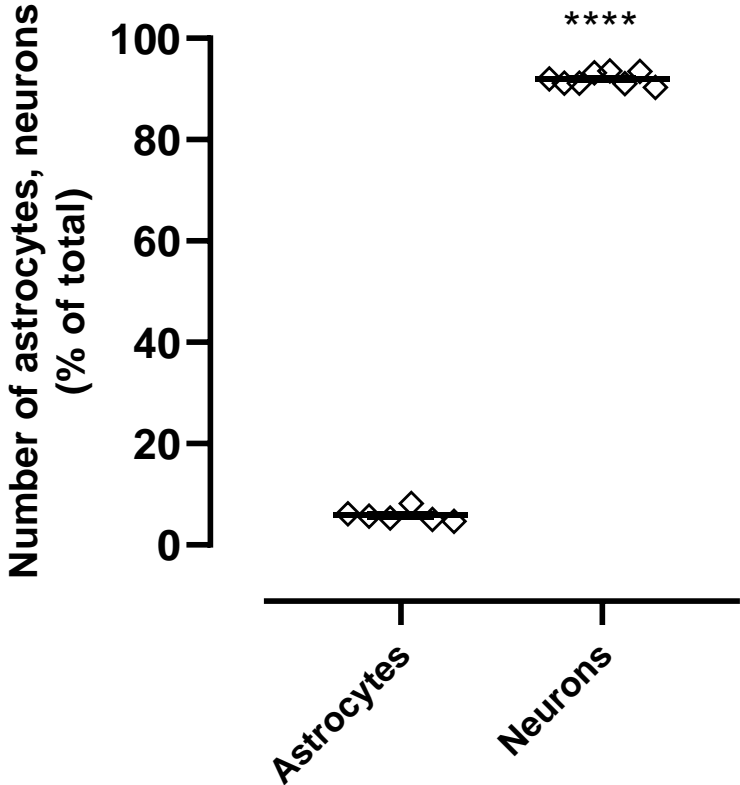

Supplement: Supplementary file 1 [file cells-13-00074-s001.zip › supplementary Figure S2.pdf]

Supplementary Figure S3

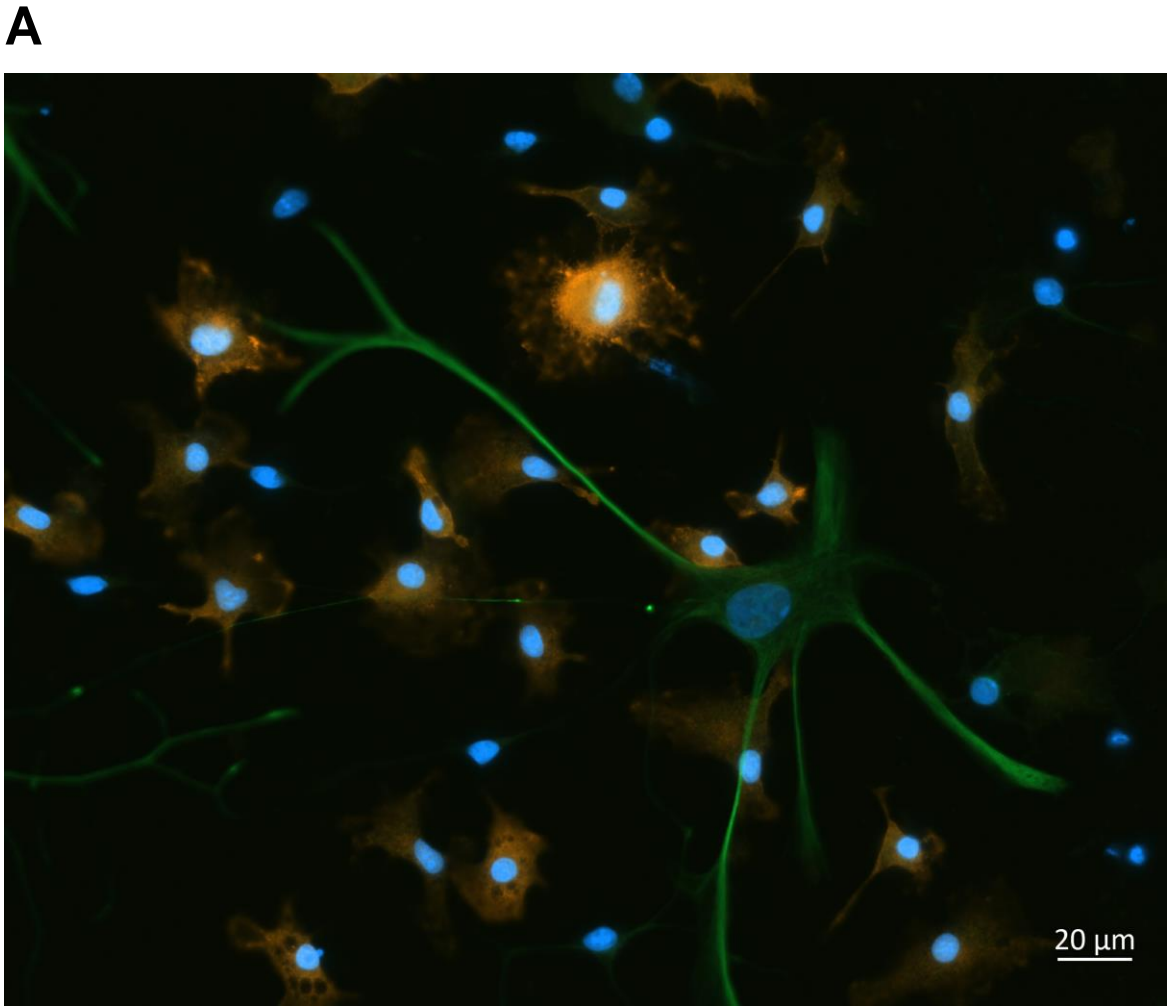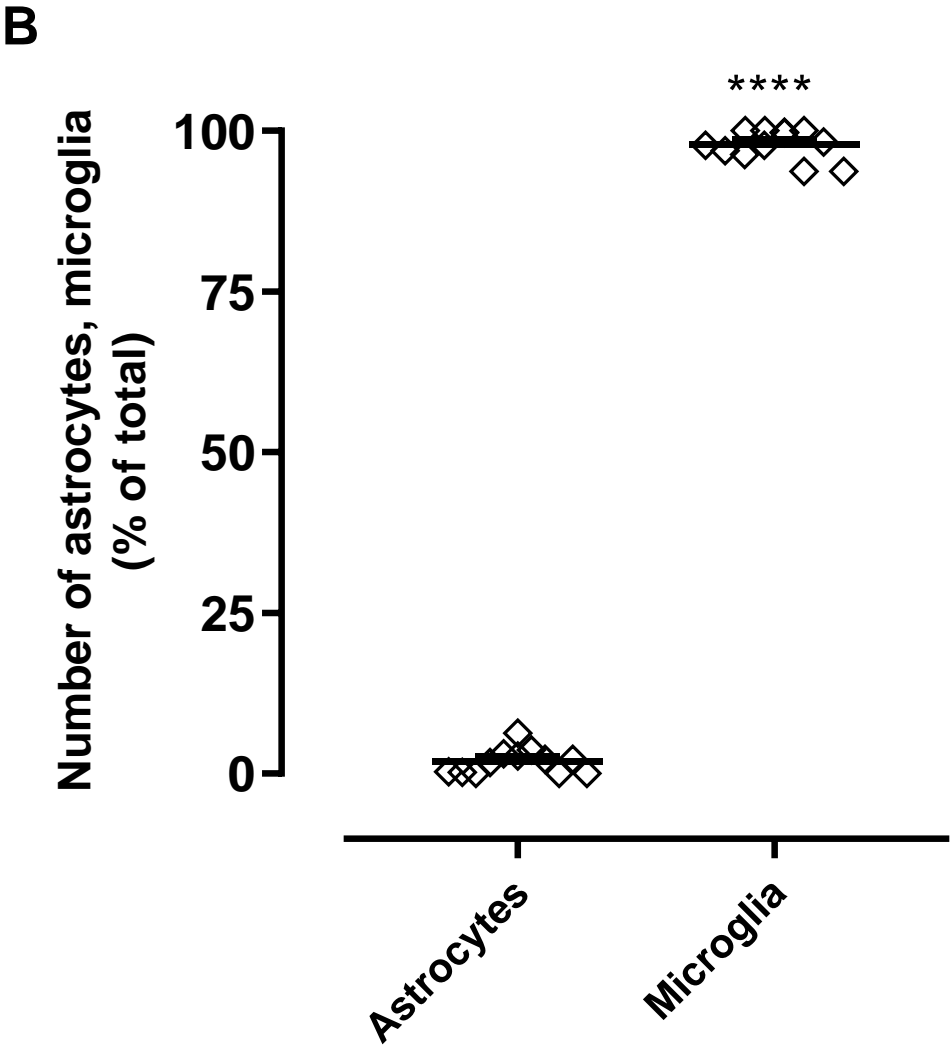

Supplement: Supplementary file 1 [file cells-13-00074-s001.zip › supplementary Figure S3.pdf]

Supplementary Figure S4

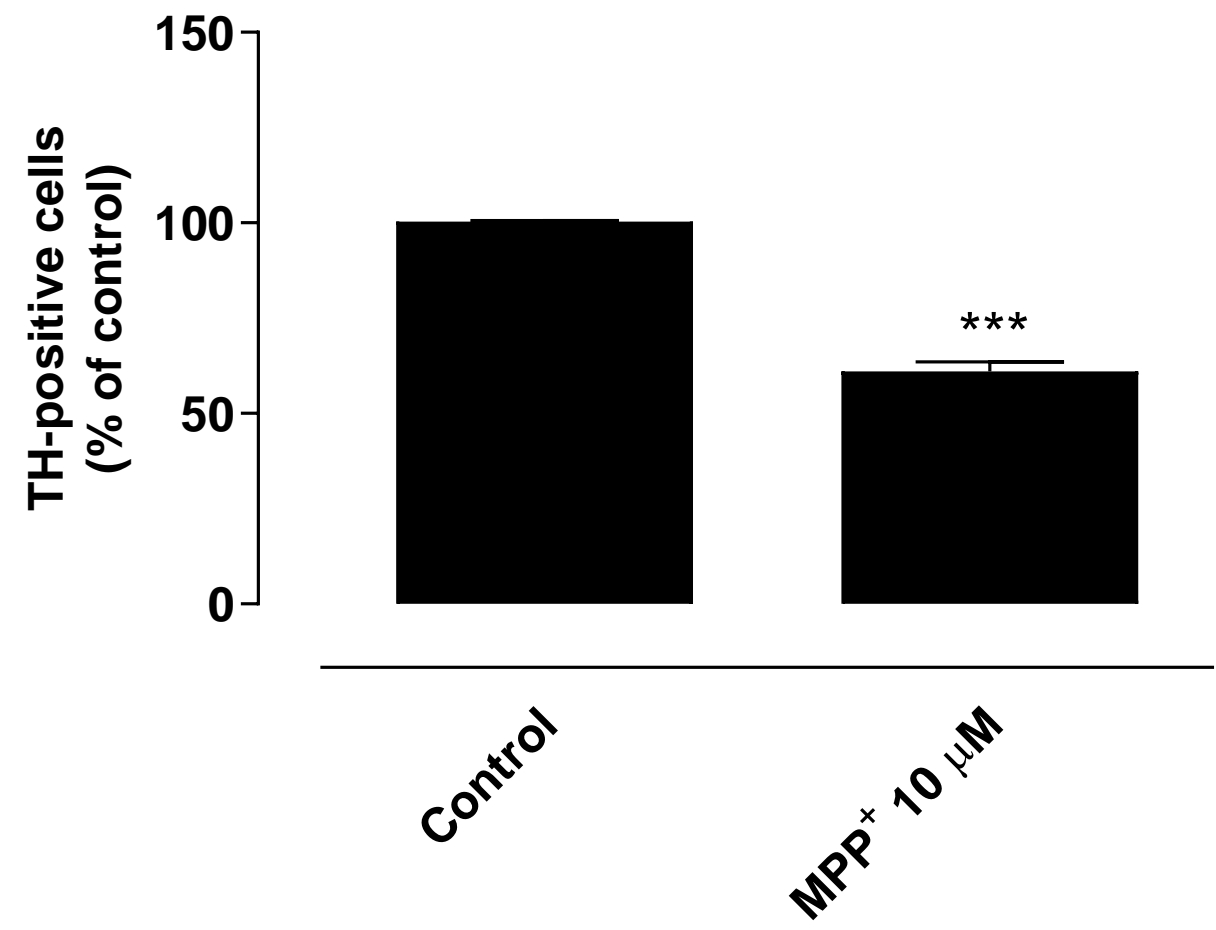

Supplement: Supplementary file 1 [file cells-13-00074-s001.zip › supplementary Figure S4.pdf]
